# Calcium channels and transporters: Roles in response to biotic and abiotic stresses

**DOI:** 10.3389/fpls.2022.964059

**Published:** 2022-09-08

**Authors:** Chang-Jin Park, Ryoung Shin

**Affiliations:** ^1^Department of Bioresources Engineering, Sejong University, Seoul, South Korea; ^2^RIKEN Center for Sustainable Resource Science, Yokohama, Japan

**Keywords:** abiotic stress, biotic stress, calcium, Ca^2+^ influx, Ca^2+^ efflux, channels, transporters

## Abstract

Calcium (Ca^2+^) serves as a ubiquitous second messenger by mediating various signaling pathways and responding to numerous environmental conditions in eukaryotes. Therefore, plant cells have developed complex mechanisms of Ca^2+^ communication across the membrane, receiving the message from their surroundings and transducing the information into cells and organelles. A wide range of biotic and abiotic stresses cause the increase in [Ca^2+^]_cyt_ as a result of the Ca^2+^ influx permitted by membrane-localized Ca^2+^ permeable cation channels such as CYCLIC NUCLEOTIDE-GATE CHANNELs (CNGCs), and voltage-dependent HYPERPOLARIZATION-ACTIVATED CALCIUM^2+^ PERMEABLE CHANNELs (HACCs), as well as GLUTAMATE RECEPTOR-LIKE RECEPTORs (GLRs) and TWO-PORE CHANNELs (TPCs). Recently, resistosomes formed by some NUCLEOTIDE-BINDING LEUCINE-RICH REPEAT RECEPTORs (NLRs) are also proposed as a new type of Ca^2+^ permeable cation channels. On the contrary, some Ca^2+^ transporting membrane proteins, mainly Ca^2+^-ATPase and Ca^2+^/H^+^ exchangers, are involved in Ca^2+^ efflux for removal of the excessive [Ca^2+^]_cyt_ in order to maintain the Ca^2+^ homeostasis in cells. The Ca^2+^ efflux mechanisms mediate the wide ranges of cellular activities responding to external and internal stimuli. In this review, we will summarize and discuss the recent discoveries of various membrane proteins involved in Ca^2+^ influx and efflux which play an essential role in fine-tuning the processing of information for plant responses to abiotic and biotic stresses.

## Introduction

Plants often survive in constantly changing environments that are stressful for their normal growth. To recognize and cope with these stress conditions caused by various biotic and abiotic factors, plants have evolved sophisticated mechanisms to use intracellular signaling molecules as second messengers in alerting cells (Lecourieux et al., 2006; Zhang et al., 2014). The calcium ion (Ca^2+^) acts as an important secondary signaling molecule and plays critical roles in many biological processes across organisms. Exposure to various sources of abiotic stresses, including heat, metals, salt, wounding, cold, and hypoxia, cause the increase in cytosolic Ca^2+^ ([Ca^2+^]_cyt_). Activation of PATTERN RECOGNITION RECEPTORs (PRRs) by extracellular patterns and NUCLEOTIDE-BINDING LEUCINE-RICH REPEAT (NLR) receptors by cytosolic pathogenic effectors induces PATTERN-TRIGGERED IMMUNITY (PTI) and EFFECTOR-TRIGGERED IMMUNITY (ETI), respectively. Either layer of the immune system also evokes cytosolic Ca^2+^ signals as a conserved overlapping cell signaling event. The magnitude and pattern of the increases of [Ca^2+^]_cyt_ vary upon the different stresses (Bose et al., 2011), and the distinct specificities of Ca^2+^ signals are generally referred to as a Ca^2+^ signature. Ca^2+^ channels are involved in the removal of the excessive [Ca^2+^]_cyt_ and the [Ca^2+^]_cyt_ removed from cytosol by Ca^2+^-ATPases is mainly stored in the endoplasmic reticulum (ER).

The advance of genomics and molecular biology has accelerated and enabled genome-wide studies, which have resulted in the identification of various Ca^2+^ channels and pumps. Recent advances in Ca^2+^ signal analysis with high-throughput genetics screens has facilitated the validation of the numbers of Ca^2+^ channels and pumps which are essential contributors to the Ca^2+^ signature. However, the specific functions of each member of the Ca^2+^ channels and pumps in stress signaling are only beginning to emerge. In addition, as more research progresses on Ca^2+^ signaling upon exposure to diverse stresses, additional unanswered questions continued to be revealed. In this review, we discuss the recent studies of Ca^2+^ channels and pumps which have focused on elucidating their functional roles in plant stresses.

## Ca^2+^ influx

### Cyclic nucleotide-gated channels

Plant CYCLIC NUCLEOTIDE-GATED CHANNELs (CNGCs), first discovered in barley (Schuurink et al., 1998), are nonspecific Ca^2+^-permeable cation channels (Sanders et al., 2002; Ali et al., 2007; Jha et al., 2016; Duszyn et al., 2019). They have been predicted as multiple genes in many plant species; *Arabidopsis thaliana* (Arabidopsis) has 20 CNGCs (Schuurink et al., 1998) and *Oryza sativa* (rice) has 16 (Nawaz et al., 2014). CNGCs are members of the “P-loop” superfamily of cation channels present in all prokaryotic and eukaryotic cells (Ward et al., 2009). The channel structure is formed by four subunits, each of which has six transmembrane domains including a positively charged transmembrane domain, a pore region (P-loop) between the fifth and sixth domains, and a CYCLIC NUCLEOTIDE BINDING DOMAIN (CNBD; Zelman et al., 2012; Duszyn et al., 2019). The CNBD contains a PHOSPHATE BINDING CASSETTE (PBC) and hinge region with a CALMODULIN-BINDING DOMAIN (CaMBD) at its C-terminus. Most CNGCs have been demonstrated to localize to the plasma membrane (Duszyn et al., 2019). However, a seemingly contradictory findings of mitochondrial, nuclear, and vacuolar membrane localization of CNGCs has been also reported (Duszyn et al., 2019). It is plausible that the contrasting observations of their locations could be caused by extensive formation of different CNGC heterotetramers.

CALMODULIN-BINDING DOMAIN is located in a site overlapping with the C-terminal α-helix of the CNBD, which allows CALMODULIN (CaM) to compete with cyclic nucleotide monophosphate (cGMP/cAMP) as a ligand in allosteric gating of channel conductance (Kaplan et al., 2007). However, in contrast to the previously proposed competitive ligand model, some CNGCs were found to carry more CaM-BINDING SITE (CaMBS) than initially reported (Fischer et al., 2013, 2017; DeFalco et al., 2016a,b). AtCNGC20 binds CaM *via* “ISOLEUCINE-GLUTAMINE” (IQ) domains adjacent to but not overlapping an α-helix in the CNBD (Fischer et al., 2013). Additionally, AtCNGC12 also contains multiple CaMBSs at cytosolic N and C termini, and apoCaM lacking bound Ca^2+^ interacts with a conserved IQ domain, while Ca^2+^/CaM binds additional N- and C-terminal motifs with different affinities (DeFalco et al., 2016a).

**Figure 1:**
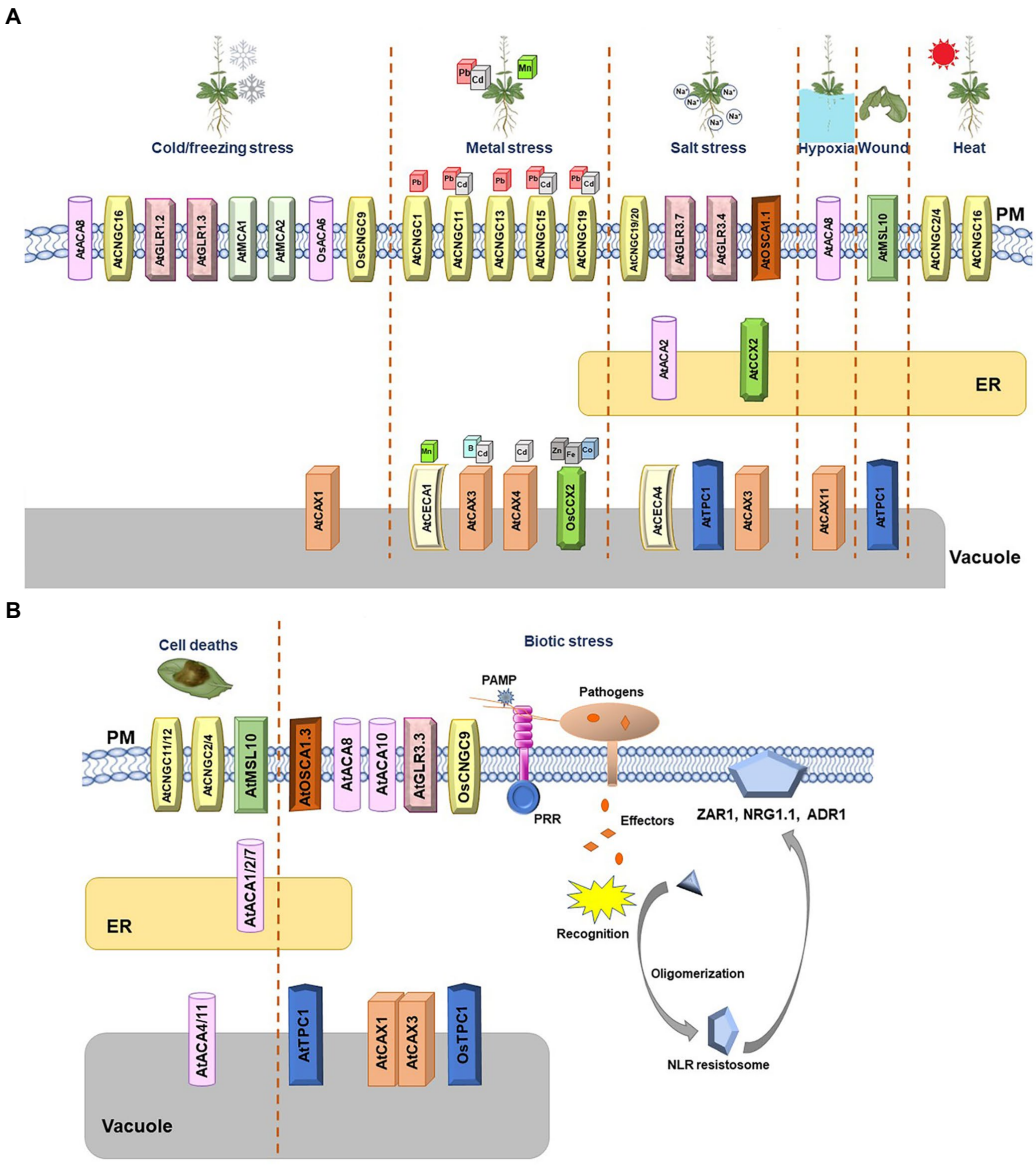
Arabidopsis and rice Ca^2+^ channels and transporters involved in diverse stresses. **(A)** Schematic summary of Arabidopsis and rice Ca^2+^ channels involved in abiotic stresses. The Ca^2+^ channels whose function were validated in Arabidopsis and rice are shown. Cold/freezing, metal, salt, hypoxia, wound, and heat stresses are depicted. When two channels create a complex and function as one unit, they are marked together. **(B)** Schematic summary of Arabidopsis and rice Ca^2+^ channels involved in biotic stress and cell death. The calcium channels whose function were validated in Arabidopsis and rice are shown. Transporters belonging to the same family are marked with the same geometric shapes. When two or more channels create a complex and function as one unit, they are marked together.

Transient but robust changes in intracellular Ca^2+^ concentration upon pathogen infection have been reported as vital early signaling to induce defense responses (Lecourieux et al., 2006; Zhang et al., 2014). Arabidopsis null mutants, *atcngc2* and *atcngc4* (also called *defense, no death 1* and *2*), displayed impaired cell death upon exposure to avirulent bacteria, indicating that AtCNGC2 and AtCNGC4-mediated Ca^2+^ signaling is critical for plant disease resistance (Yu et al., 1998; Jurkowski et al., 2004) (Figure 1). Additionally, they have nearly identical phenotypes, including constitutively activated defense response, elevated levels of salicylic acid (SA), and increased expression of *PATHOGEN RESISTANCE* (*PR*) genes (Clough et al., 2000; Jurkowski et al., 2004). It was reported that AtCNGC2 conducts Ca^2+^ into cells and the *dnd1* mutant (*atcngc2*) without functional AtCNGC2 lacks this cell membrane Ca^2+^ current and does not display cell death (Ali et al., 2007). Bimolecular fluorescence complementation analyses in *Nicotiana benthamiana* support the hypothesis that AtCNGC2 and AtCNGC4 are likely part of the same heterotetrameric channel complex (Chin et al., 2013). Ca^2+^ accumulation in response to H_2_O_2_ were reduced in *atcngc2* and *atcngc4* mutants (Tian et al., 2019) and PATHOGEN-ASSOCIATED MOLECULAR PATTERN (PAMP)-induced REACTIVE OXYGEN SPECIES (ROS) production was impaired in *atcngc2* mutant (Ma et al., 2009), suggesting mutual interplay of Ca^2+^ channels and ROS signaling in plant immune response. The Arabidopsis gain-of function mutant, *CONSTITUTIVE EXPRESSER OF PR GENES 22* (*cpr22*), was identified as the fusion of two tandemly repeated *AtCNGC11* and *AtCNGC12* genes (Yoshioka et al., 2001, 2006). The *cpr22* mutant shares a similar phenotype with *dnd1* and *dnd2*, displaying elevated levels of SA and increased expression of *PR* genes, with the exception that is *cpr22* induced upon cell death.

The rice OsCNGC9 mutant, *oscngc9* (*cds1*, cell death and susceptible to blast 1), displayed a lesion mimic phenotype and impaired resistance to *Magnaporthe oryzae* (Wang et al., 2019b). Rice receptor-like cytoplasmic kinase 185, inducing PTI, physically interacts with and phosphorylates OsCNGC9 to activate its channel activity and Ca^2+^ influx (Wang et al., 2019b). The expression patterns of many rice *CNGCs* were up- or downregulated upon various pathogens, suggesting their functional involvement as defense-related genes. For examples, more than 10 *OsCNGC* genes were significantly upregulated in rice inoculated with *Xanthomonas oryzae* pv. *oryzae* (*Xoo*) and *Pseudomonas fuscovaginae* (Nawaz et al., 2014).

Interestingly, the *cpr22* mutant of Arabidopsis was temperature sensitive, displaying more intense cell death when grown under low temperature conditions (16°C; Chin et al., 2010; Mosher et al., 2010). The null mutants for *atcngc2* (dnd1) and *atcngc4* (dnd2) also show temperature-sensitive cell death phenotypes (Yu et al., 1998; Jurkowski et al., 2004; Finka et al., 2012). In addition, *atcngc2* mutants displayed enhanced tolerance to heat stress with higher levels of heat response protein that accumulate during the seeding stage (Katano et al., 2018). Genetic evidence identified AtCNGC16 as critical for pollen fertility under stress conditions (Tunc-Ozdemir et al., 2013). Under hot/cold and drought stresses, *atcngc16* resulted in a greater than 10-fold stress-dependent reduction in pollen fitness and seed set (Tunc-Ozdemir et al., 2013). Arabidopsis transgenic overexpressing AtCNGC19 or AtCNGC20 displayed salt tolerance and their knockout plants become more sensitive to the salt stress (Oranab et al., 2021). Some AtCNGCs involved in the uptake and transport of heavy metal ions such as Pb^2+^ and Cd^2+^. *atcngc11*, *atcngc15*, and *atcngc19* resulted in reduced Pb^2+^ and Cd^2+^ accumulation, while *atcngc1* and *atcngc13* displayed reduced Pb^2+^ accumulation but not Cd^2+^ (Moon et al., 2019). Distinct groups of AtCNGCs appear to have different characteristics regarding their roles in heavy metal uptake. In rice, OsCNGC9 conferred chilling tolerance by regulating cold-induced calcium influx and the activation of cold stress-related genes (Wang et al., 2021). Many *OsCNGC* genes were also differentially regulated upon exposure to cold stress (Nawaz et al., 2014). For example, *OsCNGC6* showed a 192-fold increase, while *OsCNGC16* showed a 2-fold decrease in response to cold stress. These findings provide functional evidence that a calcium signaling cascade mediated by CNGCs plays a role in plant acclimatization to diverse abiotic stresses.

### Nucleotide-binding leucine-rich repeat receptors

Recently, several studies showed that activated NUCLEOTIDE-BINDING LEUCINE-RICH REPEAT RECEPTORs (NLRs) containing a COILED-COIL (CC) domain or a RESISTANCE TO POWDERY MILDEW 8 (RPW8)-like CC domain function as Ca^2+^ channels to induce cell death and defense responses (Bi et al., 2021; Jacob et al., 2021). CC-NLR and RPW8-like CC-NLR are known as CNL and RNL, respectively. Arabidopsis HOPZ-ACTIVATED RESISTANCE 1 (ZAR1) represents a CNL, which is one of the best structurally characterized sensor NLR recognizing pathogen effectors (Wang et al., 2019a,b). Inactive ZAR1 exists in a preformed complex with RESISTANCE-RELATED KINASE 1 (RKS1; Wang et al., 2015). *X. campestris* type III secreted effector protein, AvrAC, uridylates receptor-like cytoplasmic kinase PBL2 resulting in PBL2^UMP^. The PBL2^UMP^ binds to the ZAR1-RKS1 complex, which induces ZAR1 conformational changes to an active pentameric state of ZAR1-RKS1-PBL2^UMP^ complex, called a resistosome (Wang et al., 2019c; Hu et al., 2020). The ZAR1 resistosome, containing an ion-conducting pore constituted by the N-terminal helix α1 domains, penetrates the plasma membrane and forms a calcium-permeable cation channel (Bi et al., 2021). The calcium influx further leads to accumulation of reactive oxygen species, activation of cell death, and defense response. Therefore, ZAR1 functions as both a sensor of pathogens and an executor of immune response as a functional Ca^2+^ channel (Wang et al., 2019c; Bi et al., 2021; Wan and He, 2021).

Another type of sensor NLR proteins containing a TOLL/INTERLEUKIN-1 RECEPTOR (TIR) domain, TNLs, have been confirmed to possess oligomerization-dependent NICOTINAMIDE ADENINE DINUCLEOTIDE (NAD) HYDROLASE (NADase) activity within their TIR domain (Horsefield et al., 2019; Wan et al., 2019). Tetramerization in the TIR domain of Arabidopsis TNL, RECOGNITION OF *PERONOSPORA PARASITICA* 1 (RPP1), creates the active site for catalysis after RPP1 recognizes and directly binds *Hyaloperonospora arabidopsidis* effector, *A. thaliana* RECOGNIZED 1 (ATR1; Ma et al., 2020; Martin et al., 2020). The oligomerization-dependent NADase activity was also proposed in *Nicotiana benthamiana* (tobacco) TNL, RECOGNITION OF *XANTHOMONAS* OUTER PROTEIN Q 1 (XopQ 1; ROQ1), after it interacts with *Xanthomonas* effector protein, XopQ 1 (Martin et al., 2020). NAD^+^ and its cleavage products are known to perform many essential cellular functions, including immune signaling and the activation of calcium channels (Lee and Zhao, 2019; Bayless and Nishimura, 2020). In Arabidopsis, TNLs require the redundant helper NLRs which transduce signals downstream from sensor NLRs (Koster et al., 2022). Helper NLRs, ACTIVATED DISEASE RESISTANCE 1 (ADR1), and N REQUIREMENT GENE 1.1 (NRG1.1) subfamilies, belong to the RNL family (Jubic et al., 2019). The mechanisms of how the helper NLRs, ADR1, and NRG1 are activated by TIR domain NADase activity is an active area of research. Auto-active AtNRG1.1 D485V and wild-type AtADR1 induce autonomous cell death in *N. benthamiana*, functioning as calcium permeable cation channels. They oligomerize, enrich in PM, and induce Ca^2+^ influx to cause cell death in *N. benthamiana* and human HeLa cells, and the cell death activity has been shown to be inhibited by Ca^2+^ channel blockers, LaCl_3_ and GdCl_3_ (Jacob et al., 2021). Therefore, it was proposed that TNL activation induces RNL-dependent Ca^2+^ influx, to initiate cell death and, likely, immune responses (Jacob et al., 2021).

So far, there is no direct evidence that the Ca^2+^-permeable cation channels consisting of CNLs and RNLs are involved in abiotic stresses. However, the Ca^2+^ influx caused by NLR activation induces massive transcriptional reprogramming toward abiotic stress response, including apetala2/ethylene responsive factor, basic helix–loop–helix, MYB, WRKY, basic leucine zipper, and CaM-binding transcription activator families (Jacob et al., 2018; Ng et al., 2018). Further validation should be undertaken to determine the possible overlapping of NLR-mediated Ca^2+^ influx in transcriptional responses between pathogen perception and abiotic stress.

### Glutamate receptor like receptors

Mammalian IONOTROPIC GLUTAMATE RECEPTORs (iGluRs) are known to mediate the majority of excitatory neurotransmission in the central nervous system (Wisden and Seeburg, 1993). Functionally, iGluRs are GLUTAMATE (Glu)-gated cation channels that are selective for Na^+^, K^+^, and Ca^2+^ ions. Plant GLUTAMATE RECEPTOR-LIKE RECEPTORs (GLRs) have a high similarity to their animal counterparts in respect to their channel structure, ligand binding domain, and amino acid sequence (Lacombe et al., 2001). Arabidopsis has 20 GLRs and most plants possess more than 10 GLRs in their genome (Lacombe et al., 2001; Sanders et al., 2002; Kwaaitaal et al., 2011; Tapken et al., 2013; Demidchik et al., 2018; Qiu et al., 2020). In contrast to the ligand specificity of mammalian iGluRs that are mainly gated by Glu, Arabidopsis GLRs, AtGLR1.4, AtGLR3.3, and AtGLR3.4, are gated by a broad spectrum of amino acids, at least 12 out of 20 proteinogenic amino acids and the triple reduced glutathione (Qi et al., 2006; Tapken et al., 2013; Forde and Roberts, 2014). It is hypothesized that this broader ligand specificity of GLRs is caused by differences in the sequence of their pore regions (Forde and Roberts, 2014). Heterologous expression in *Xenopus* oocytes revealed that AtGLR1.4, like mammalian iGluRs, functioned as a nonselective Ca^2+^-permeable cation channel (Tapken et al., 2013), but that AtGLR3.4 was highly selective for Ca^2+^ (Vincill et al., 2012). Biological functions of plant GLRs have been reported in many aspects including biotic and abiotic stress responses (Meyerhoff et al., 2005; Kang et al., 2006; Kwaaitaal et al., 2011; Vincill et al., 2012; Li et al., 2013; Forde and Roberts, 2014).

Possible involvement of plant GLRs in defense responses was suggested in several early reports. Overexpression of *Raphanus Sativus* (radish) *GLR* in Arabidopsis triggered greater Ca^2+^ influx after glutamate treatment and conferred enhanced resistance to a necrotic fungal pathogen, *Botrytis cinerea* (Kang et al., 2006). Later, using the experiments with antagonists of mammalian iGluRs, initiation of defense responses upon several PAMPs, FLAGELLIN 22 (flg22), ELONGATION FACTOR TU 18 (elf18), and fungal chitin, involved apoplastic Ca^2+^ influx *via* iGluR-like channels, suggesting that GLRs are related to the induction of defense response after PAMP recognition (Kwaaitaal et al., 2011). In addition, loss-of-function *atglr3.3* mutants showed decreased expression of defense-related genes and increased susceptibility to the bacterial pathogen *Pseudomonas syringae* pv *tomato* DC3000 (Li et al., 2013). Genetic experiments performed with different *atglr* T-DNA mutants, *atglr3.1*, *atglr3.3*, *atglr3.4*, *atglr3.5*, and *atglr*3.7, concluded that the *atglr3.3* mutant was more susceptible to *H. arabidopsidis* and treatment of GLR antagonist compromised resistance (Manzoor et al., 2013).

AtGLR3.4 and AtGLR3.7 are involved in the regulation of seed germination with NaCl stress (Cheng et al., 2016, 2018). Recently, AtGLR3.7 was shown to be phosphorylated by a CALCIUM-DEPENDENT PROTEIN KINASE (CDPK), and its interaction with 14–3-3 proteins participates in the regulation of cytosolic Ca^2+^ concentration under salt stress (Wang et al., 2019e). The perception of cold stress by the plasma membrane can activate Ca^2+^ channels and triggers Ca^2+^-mediated signaling pathways to respond and adapt to cold stress (Ding et al., 2019; Qiu et al., 2020). The expression of *AtGLR3.4* was stimulated by exposure to cold, touch, and osmotic stress in an ABA-independent manner, but dependent upon Ca^2+^ (Meyerhoff et al., 2005). An increase in [Ca^2+^]_cyt_ triggered by cold treatment was blocked by GLRs antagonists, 6,7-dinitroquinoxaline-2,3-dione and 6-cyano-7-nitroquinoxaline-2,3-dione, suggesting that AtGLR3.4 plays a very important role in the Ca^2+^-mediated signaling transmission of cold stress. Overexpression of *AtGLR1.2* or *AtGLR1.3* improved the tolerance of mutants to cold stress by synthesizing endogenous jasmonic acid (JA) and their mutants became more sensitive to cold the stress (Zheng et al., 2018).

### Two-pore channel

In addition to the apoplast, internal cellular compartments such as the vacuole contribute to the rise in [Ca^2+^]_cyt_ (Xu et al., 2022). In the vacuolar membrane, TWO-PORE CHANNEL (TPC) functions as a nonselective cation channel co-regulated by voltage and Ca^2+^, and generates a slow vacuolar current (Furuichi et al., 2001; Ye et al., 2021). Genomic analysis indicated that there is a single *TPC1* gene in Arabidopsis (Peiter et al., 2005) and rice. Arabidopsis TPC1 is the well-studied plant TPC and is activated by the membrane depolarization and [Ca^2+^]_cyt_ but inhibited by [Ca^2+^]_vac_ (Dadacz-Narloch et al., 2011; Schulze et al., 2011). AtTPC1 forms a homodimer, where each subunit consists of two homologous six-transmembrane segment domains, therefore equivalent to a classical voltage-gated ion channel with four voltage-sensing domains and one pore domain (Guo et al., 2016; Ye et al., 2021). There are two EF-hand motifs in the cytosolic linker part of AtTPC1; Ca^2+^ binding at EF hand 1 appears to play a structural role and Ca^2+^ binding at EF hand 2 is central for Ca^2+^ activation (Schulze et al., 2011; Guo et al., 2016).

A few studies provided functional evidence to demonstrate that plant TPCs are implicated in plant immunity. Rice TPC1 (OsTPC1), localized at vacuolar membrane, functions as a Ca^2+^-permeable cation channel involved in the regulation of growth and development (Kurusu et al., 2004, 2012). A later study reported that OsTPC1 plays a positive role in elicitor-induced defense gene expression, oxidative burst, MAP kinase activation, and cell death (Kurusu et al., 2005). In Arabidopsis, the AtTPC1 gain-of-function mutant *fou2* was found to possess enhanced resistance to *B. cinerea*, accompanied with increased JA accumulation (Bonaventure et al., 2007). However, in a later study, Ca^2+^ responses in *AtTPC1*-overexpressing Arabidopsis and *attpc1-2* knockout mutants did not display any alteration in the stimulus-induced Ca^2+^ signals including abiotic stresses and PAMPs, elf18 and flg22 (Ranf et al., 2008). Therefore, it is possible that AtTPC1 is not a major player in the defense response, at least in Arabidopsis (Moeder et al., 2019; Xu et al., 2022). AtTPC1 is also known to be involved in the systemic spread of both the wound- and NaCl-driven systemic [Ca^2+^]_cyt_ increases (Choi et al., 2014, 2017; Kiep et al., 2015). In the recently proposed systemic communication pathways mediated by Ca^2+^ and ROS (Johns et al., 2021), AtTPC1 was found to be involved in the release of Ca^2+^from the vacuole, functioning as a Ca^2+^ amplifier for the initial increases in [Ca^2+^]_cyt_. The resulting elevated [Ca^2+^]_cyt_ could then further trigger RESPIRATORY BURST OXIDASE HOMOLOG (RBOH) activation for additional production of apoplastic ROS (Choi et al., 2014). A feed-forward loop between Ca^2+^ and ROS results in the propagation of the signal from cell to cell (Johns et al., 2021), suggesting the potential contribution of AtTPC1 to whole-plant stress tolerance.

### Depolarization-activated Ca^2+^ permeable channels and hyperpolarization-activated Ca^2+^ permeable channels

The particular calcium conductance mediated by DEPOLARIZATION-ACTIVATED Ca^2+^ CHANNELs (DACCs) have been found in various plant species including Arabidopsis, tobacco, and *Zea mays* (maize), although DACCs in plants have not been associated with any gene yet (Demidchik et al., 2018). Several pharmaceutical approaches using the DACC channel blockers such as nifedipine showed that abiotic stresses result in a depolarization of the plasma membranes by DACCs, followed by an increase in [Ca^2+^]_cyt_ (Crotty et al., 1996; Thion et al., 1998; Lhuissier et al., 2001; Miedema et al., 2001, 2008; Okazaki et al., 2002; Carpaneto et al., 2007; McAinsh and Pittman, 2009; White, 2009; Seifikalhor et al., 2019). DACCs are more responsive to a short and transient Ca^2+^ influx triggered by exposure to acute stress stimuli such as cold and cadmium stresses (Carpaneto et al., 2007; White, 2009; Wilkins et al., 2016).

In plants, HYPERPOLARIZATION-ACTIVATED Ca^2+^ CHANNELs (HACCs) were first found in *Solanum lycopersicum* (tomato) responding to fungal infection (Gelli and Blumwald, 1997; Manohar et al., 2011b). A primary role of HACCs is to a sustain Ca^2+^ influx such as guard cell signaling and root hair elongation (Miedema et al., 2001, 2008; Mortimer et al., 2008; Swarbreck et al., 2013; Tang and Luan, 2017). In guard cells, HACC activities were confirmed to be activated by ABA and ROS subsequent to an increase in [Ca^2+^]_cyt_. Additionally, HACC conductance is further stimulated by extracellular ATP in guard cells and pollen plasma membranes (Demidchik et al., 2009; Wu et al., 2018; Wang et al., 2019d). In the root epidermis, HACC functions downstream of the plasma membrane RBOHC and contributes to net [Ca^2+^]_cyt_ influx in the root zone (Demidchik et al., 2009, 2011; Shang et al., 2009; Wang et al., 2019d).

Annexins are membrane binding proteins that can form Ca^2+^-permeable conductance *in vitro*. Plasma membrane-localized Arabidopsis ANNEXIN1 (AtANN1) mediated Ca^2+^ influx in root epidermal cells, which is activated by extracellular ^•^OH and H_2_O_2_ (Demidchik et al., 2010; Richards et al., 2014). In addition, the AtANN1 mutant, *atann1* was found to lack root hairs and ^•^OH-activated Ca^2+^- and K^+^-permeable conductance (Demidchik et al., 2010). Furthermore, the *atann1* was also found to lack an induction of salt-induced transcripts. Salt stress and cell death often leads to an increase in extracellular ^•^OH. Collectively, these findings support the concept that AtANN1 functions as a Ca^2+^-permeable transporter link between stress-induced ROS (^•^OH) and [Ca^2+^]_cyt_ (Lee et al., 2004; Shang et al., 2009; Laohavisit et al., 2012, 2013; Richards et al., 2014; Kurusu et al., 2015; Wilkins et al., 2016). In addition, AtANN1 would function together with AtCNGC5, AtCNGC6, and AtCNGC9 for root hair growth (Tan et al., 2020), although further experimental evidence is still needed.

### Mechanosensitive-like channels

Plant cells sense mechanical stimuli in nature, such as touch, gravity, and the stretching of membranes. MECHANOSENSITIVE (MS) ion channels that serve to sense and respond to changes in membrane tension have been known to directly couple mechanical stimuli to ion flux (Peyronnet et al., 2014; Basu and Haswell, 2017). There are a few plant MS channel families that have been reported as Ca^2+^ channels (Basu and Haswell, 2017). One of them is ECHANOSENSITIVE CHANNEL OF SMALL CONDUCTANCE (MSCS)-LIKE CHANNELs (MSLs). It has been predicted that there are 10 and five *MSL* genes in Arabidopsis and rice, respectively (Haswell, 2007; Saddhe and Kumar, 2015). Arabidopsis MSL10 was shown to have a plant-specific N-terminal domain which is capable of inducing cell death in a phosphorylation-dependent manner (Veley et al., 2014). A gain-of-function mutation in *AtMSL10* triggered cell death and wound-induced hyperaccumulation of JA (Zou et al., 2016). Recently, it has been reported that AtMSL10 functions as a phospho-regulated membrane-based sensor that connects the perception of cell swelling to a downstream signaling cascade and cell death (Basu and Haswell, 2020). Finally, it was revealed that mechanical signals generated during pathogenic invasion are exploited by AtMSL10 (Basu et al., 2021). Overexpression and gain-of-function mutants of *AtMSL10* exhibited reduced susceptibility to infection by *P. syringae* pv. *tomato* DC3000 and showed an accelerated induction of Arabidopsis PATHOGENESIS-RELATED PROTEIN 1 (*AtPR1*) expression compared to wild-type plants, indicating that mechanical signals are important.

Another MS channel family functioning as a Ca^2+^ channel is a MID1-COMPLEMENTING ACTIVITY (MCA), exhibiting 10% identity to yeast MATING-INDUCED DEATH 1 (Mid1). AtMCA1 and AtMCA2 have been identified in Arabidopsis *via* functional complementation of the yeast mutant *mid1* (Yamanaka et al., 2010). MCA proteins share certain structural features, an EF hand-like and a CC motif in the N-terminal region, and two to four putative transmembrane domains and a Cys-rich PLAC8 domain of unknown function in the C-terminal region (Galaviz-Hernandez et al., 2003; Yamanaka et al., 2010; Nishii et al., 2021). In a recent study, the cold-induced [Ca^2+^]_cyt_ increase in *atmca1* and *atmca2* mutants was markedly lower than what occurred in wild-types. Importantly, the *atmca1/2* double mutant displayed increased cold sensitivity, suggesting that AtMCA1 and AtMCA2 are functionally involved in a cold-induced elevation of [Ca^2+^]_cyt_ (Mori et al., 2018). In addition, MCAs have been reported to function in diverse cellular responses to different stresses, including osmotic sensing and cell wall damage responses (Nakagawa et al., 2007; Denness et al., 2011; Moeder et al., 2019).

### Reduced “hyperosmolarity-induced [Ca^2+^]_cyt_ increase” channels

HYPEROSMOLALITY-GATED CALCIUM-PERMEABLE CHANNELs (OSCAs) were first identified as an osmosensors in Arabidopsis (Yuan et al., 2014). After studying mutants with a low intracellular free calcium concentration under high osmotic stress, OSCA was determined to function in the perception of extracellular changes to trigger hyperosmolality-induced [Ca^2+^]_cyt_ increases. Arabidopsis contains 15 AtOSCAs which possess 9 transmembrane domains, including one cleavable transmembrane domain (Demidchik et al., 2018). Predictive analysis from the rice (*O. sativa* L. Japonica) genomic database revealed a total of 11 *OsOSCA*s genes (Li et al., 2015). Due to the complex regulation of Ca^2+^ signaling and homeostasis, the potential involvement of OSCA in specific aspects of defense regulation has been suggested (Moeder et al., 2019). A recent study revealed that AtOSCA1.3 is rapidly phosphorylated upon perception of PAMP flg22, controlling stomatal closure during immune signaling (Thor, 2019). AtOSCA1.1 is reported to be involved in sensing extracellular changes which results in a triggering of increases in hyperosmolality-induced [Ca^2+^]_cyt_ (Yuan et al., 2014).

## Ca^2+^ efflux

### Ca^2+^-ATPases

One of the major membrane protein families which are responsible for Ca^2+^ efflux, Ca^2+^-ATPases, is high affinity (Km = 0.1–2.0 μM) and low capacity Ca^2+^ transporter (Garcia Bossi et al., 2020). Its primary role is the termination of Ca^2+^-mediated signaling. The P-type Ca^2+^-ATPases are directly activated by ATP and are found in animal, fungi, as well as plants (Geisler et al., 2000a). A primary role of plant Ca^2+^-ATPases is to maintain ion homeostasis through the pumping of [Ca^2+^]_cyt_ out of the cytosol. Plant Ca^2+^-ATPases belong to either the P_2a_-type Ca^2+^-ATPases (ECA) or P_2b_-type AUTOINHIBITED CA^2+^-ATPASEs (ACAs) which have 10 transmembrane domains (Demidchik et al., 2018; Demidchik and Shabala, 2018). ECAs are mostly localized on endomembranes, but ACAs carrying an N-terminal CaM-regulated autoinhibitory domain have been confirmed to localize on plasma membranes or endomembranes (Geisler et al., 2000b; Huda et al., 2013c). There are 10 ACAs and 4 ECAs in *Arabidopsis* and 11 ACAs and 3 ECAs in rice (Sze et al., 2000; Geisler et al., 2000b; Baxter et al., 2003; Huda et al., 2013c,d). ACAs are known to exclusively transport Ca^2+^ but ECAs, however, are capable of transporting Ca^2+^ and Mn^2+^ (Baxter et al., 2003). ECAs are similar to mammal SACRO/ENDOPLASMIC RETICULUM CACLIUM ATPASE (SERCA), which are known as transporters of Ca^2+^, Mn^2+^, and Zn^2+^ (Carafoli and Brini, 2000; Wu et al., 2002). Despite this similarity, mammal ECAs are regulated by phospholamban but plant ECAs do not have phospholamban-binding sites (Baxter et al., 2003). ACAs that are similar to mammalian CaM-stimulated ATPases, and plant ACAs were confirmed to be localized on the multiple cellular position different from only plasma membrane-localized animal ACAs. Specifically, AtACA2 is localized on the ER (Harper et al., 1998), AtACA4 is vacuolar (Geisler et al., 2000b; Baxter et al., 2003), and AtACA8 resides in the plasma membrane (Bonza et al., 2000). Genome structure analysis of ACAs determined that they are divided into four subfamily members in plants (Carafoli and Brini, 2000; Sze et al., 2000; Wu et al., 2002). Arabidopsis AtECA1, AtECA2, AtECA4 and rice OsECA1, OsECA2, and OsECA4 belong to subfamily I; and OsECA3 and AtECA3 belong to subfamily II (Baxter et al., 2003).

Abiotic and biotic stress have been known to increase [Ca^2+^]_cyt_, which is subsequently followed by the accumulation of ROS, and the excessive [Ca^2+^]_cyt_ is eventually diminished from cytosol (Beffagna et al., 2005; Lecourieux et al., 2006; Mortimer et al., 2008; Laohavisit et al., 2013; Swarbreck et al., 2013; Shabala et al., 2016; Wilkins et al., 2016; Ahmadi et al., 2018; Ahmed et al., 2018; Demidchik et al., 2018; Demidchik and Shabala, 2018). Many reports have documented the contributions of plant Ca^2+^-ATPases for the removal of excessive [Ca^2+^]_cyt_ from the cytosol. The double mutant of vacuolar localized AtACA4 and AtACA11 results in a high frequency of cell death that is suppressed when plants are grown in the presence of more than 15 mM anions by decreasing SA. AtACA8 and AtACA10 were confirmed to function as positive regulators for a PAMP-triggered Ca^2+^ burst and a double knockout of AtACA4 and AtACA11, *ataca4/11* displayed SA-dependent cell death-like lesions. Similarly, tobacco plants which lacked NbCA1 exhibited enhanced cell death in response to the tobacco mosaic virus (Nemchinov et al., 2008; Boursiac et al., 2010; Huda et al., 2013c). The vacuolar ACAs have been shown to mediate a SA-dependent cell death response in plants (Boursiac et al., 2010). An ER-localized *AtACA1*, *AtACA2*, and *AtACA7* triple mutant, *ataca1/2/7*, was confirmed to have reduced pollen fertility and smaller rosette size. These genes were suggested to be functionally redundant since each of the three genes could complement the defective phenotype. Similar to the like *ataca4/11* mutant, the *ataca1/2/7* triple mutant also displays cell death but to a lesser extent. In addition, the expression of *NahG* encoding salicylate hydroxylase was found to attenuate the cell death of the *ataca1/2/7* triple mutant (Resentini et al., 2021). AtACA8, 10, 12, and 13 constitute a complex with an Arabidopsis PRR redundantly and regulate plant immune response *via* the removal of excessive Ca^2+^ from the cytosol, and subsequently control the PAMP-triggered Ca^2+^ signaling (Frei Dit Frey et al., 2012; Yu et al., 2018; Tian et al., 2020). The elevations of [Ca^2+^]_cyt_ during cell death are essential for defense response, including oxidative burst (Atkinson et al., 1990; Levine et al., 1996; Bose et al., 2011). Cell death has been shown to be inhibited by Ca^2+^ channel blockers *via* blocking the elevation of Ca^2+^ influx resultant from the initiation of cell death. After mimicking cell death, [Ca^2+^]_cyt_ levels were found to dramatically and rapidly decrease, resulting in the prevention of any damage from oxidative stress. Ca^2+^-ATPases are known to partially mediate this aforementioned Ca^2+^ efflux (Atkinson et al., 1990; Levine et al., 1996; Nemchinov et al., 2008; Boursiac et al., 2010; Pottosin et al., 2014). In tobacco, Ca^2+^-ATPases are involved in the removal of excessive Ca^2+^ that was caused by Potato Virus X (PVX)-induced acquired resistance (Shabala et al., 2011b). Ca^2+^ efflux that mediated by Ca^2+^-ATPases contributed to the process of PVX-induced resistance to oxidative stress in tobacco (Shabala et al., 2011a). These reports support that Ca^2+^-ATPases play important roles in abiotic and biotic stress through adjusting [Ca^2+^]_cyt_ levels.

A lack of *AtACA2* and *AtACA4* resulted in an increase in salt sensitivity but their overexpression led to salt tolerance in yeast *Saccharomyces cerevisiae*. The function of AtACA2 was shown to restore [Ca^2+^]_cyt_ that was induced by salt stress in yeast (Schiott et al., 2004; Anil et al., 2008; Seifikalhor et al., 2019). In plants, the expression of *AtACA8* and *AtACA9*, but not *AtACA10*, were confirmed to be up-regulated by ABA. When exposed to cold stress, contrasting expression patterns were observed with *AtACA8* showing increased expression to cold stress, whereas, *AtACA10* expression was decreased. The loss-of-function of *AtACA8* resulted in a higher Ca^2+^ accumulation in roots during hypoxia (Schiott et al., 2004; Cerana et al., 2006). The N-terminal modification of AtACA4 was found to be associated with increased salt tolerance (Geisler et al., 2000b). Collectively, these findings suggest that ACAs induced by abiotic stresses are mainly involved in the responses to abiotic stresses *via* the removal of [Ca^2+^]_cyt_ which results from these abiotic stresses. AtECA4-mediated recycling of proteins from the endosome to the plasma membrane plays a key role in salt-induced ROS accumulation independent from PAMP flg22-induced ROS accumulation (Lee et al., 2021). ER-localized AtECA1 restored yeast growth on a high Mn^2+^ and Zn^2+^ background (Sze et al., 2000). Additionally, AtECA1 also controlled plant growth in Ca^2+^ deficient or Mn^2+^ toxic conditions (Wu et al., 2002). Multiple rice ACAs are involved in abiotic stress, including OsACA4 in salt stress (Yamada et al., 2014). In tobacco, overexpression of *OsACA6* led to increased abiotic stress tolerance toward drought, cold, Cd, and UV (Huda et al., 2013a; Kamrul Huda et al., 2014; Shukla et al., 2014). The promoter sequence of rice plasma membrane-localized Ca^2+^-ATPase has several cis-elements which respond to various abiotic stresses including ABA, light, wounding, dehydration, cold and heat (Huda et al., 2013b). The increased Ca^2+^ fluxes and ROS caused by hyperosmotic and hypoosmotic stress were attenuated by eosin yellow which is a selective inhibitor of plasma membrane Ca^2+^-ATPases (Beffagna et al., 2005).

Overexpression of *Medicago sativa* (Alfalfa) ACAs, *MsRCI2s* resulted in increased tolerance to alkaline and salt stress (Li et al., 2021). Expression of a plasma membrane-localized SOYBEAN CA^2+^-ATPASE (*SCA1*) was rapidly induced by NaCl or the fungal elicitor treatment, but not by KCl or mannitol treatment. The regulation of SCA1 activity was shown to be Ca^2+^-dependent and CaM-binding-dependent (Chung et al., 2000). In chilling-sensitive wheat, a 2°C chilling treatment led to an increase in the [Ca^2+^]_cyt_ level and a decrease in Ca^2+^-ATPase activity. On the other hand, in chilling tolerant winter wheat, however, Ca^2+^ level was restored and Ca^2+^-ATPase activity was maintained (Jian et al., 1999). Expression of *Physcomitrella patens* (moss) ACA, PCA1, is localized within small vacuoles, and was shown to be up-regulated by drought, salt, and abscisic acid. A knockout of *PCA1* resulted in alteration of the responses to [Ca^2+^]_cyt_, which in turn altered the expression of stress-induced genes and interfered with the tolerance response to abiotic stress (Qudeimat et al., 2008). Barley varieties that are tolerant to Al are characterized by increased Ca^2+^-ATPase activities (Ahmed et al., 2018). Boron-starvation led to an increase in root hair growth, and up-regulated expression of ACAs (*AtACA10, AtACA11, AtACA12*, and *AtACA13*) in Arabidopsis roots (Gonzalez-Fontes et al., 2013, 2014; Quiles-Pando et al., 2013; Wilkins et al., 2016). As a consequence of boron deficiency, Ca^2+^-ATPase activity was induced by NO and functioned to alleviate Fe^2+^ deficiency in peanut (Song et al., 2018).

### Ca^2+^/cation antiporter and Ca^2+^/H^+^ Exchanger

CALCIUM/CATION ANTIPORTERs (CaCAs), especially CA^2+^/H^+^ EXCHANGERs (CAXs), largely contribute to Ca^2+^ homeostasis in plant cells and they are activated by the transport of counter cations. Three subfamilies of CaCAs, NA^+^/CA^2+^ EXCHANGERs (NCXs), CATION/Ca^2+^ EXCHANGERs (CCXs) and CAX, were confirmed to exist in land plants (Bose et al., 2011; Emery et al., 2012; Mao et al., 2021). Until recently, it was thought that NCXs existed only in animal cells. However, a bioinformatic genome analysis has suggested that a putative *NCX* gene exists within the plant genome. Plant CAXs are comprised of 11 transmembrane domains, an acidic motif between sixth and seventh transmembrane domains, a N-terminal autoinhibitory domain, and a Ca^2+^-specific binding domain (Shigaki and Hirschi, 2006; Manohar et al., 2011a; Demidchik et al., 2018). In Arabidopsis, an NCX-LIKE (AtNCL) protein was confirmed to have Ca^2+^ binding activity and its expression was upregulated by salt stress. Furthermore, the mutant plant of AtNCL exhibited reduced sensitivity to salt stress (Shigaki and Hirschi, 2006). This finding suggested that AtNCL mediated the Ca^2+^ homeostasis in the presence of high levels of available Na^+^ (Wang et al., 2012). The functions of plant CCXs are not well known at this time. Recently, an Arabidopsis AtCCX1 was revealed to be involved in leaf senescence and exhibits an affinity to Ca^2+^ (Conn et al., 2011; Li et al., 2016). Although AtCCX3, AtCCX4, and AtCCX5 were suggested to have affinity to K^+^, Na^+^, or Mn^2+^, there is no evidence that they have an affinity to Ca^2+^ (Morris et al., 2008; Zhang et al., 2011; Corso et al., 2018). The ER-localized Arabidopsis CCX2, AtCCX2, has been shown to adjust osmotic stress *via* a direct control of Ca^2+^ fluxes between the cytosol and the ER. *AtCCX2* knockout plants showed a decreased [Ca^2+^]_cyt_ and increased ER Ca^2+^, and exhibited reduced tolerance to osmotic stress (Corso et al., 2018).

CA^2+^/H^+^ EXCHANGERs are low affinity (Km = 10–15 μM) and high capacity Ca^2+^ transport. CAXs restore [Ca^2+^]_cyt_ levels *via* removal of [Ca^2+^]_cyt_ (Sze et al., 2000; Bose et al., 2011). Arabidopsis CAX3, *AtCAX3* expression was induced by PAMP flg22 and mechanical wounding (Ecker and Davis, 1987; Hocking et al., 2017). Co-expression of *AtCAX1/AtCAX3* was found in mesophyll cells during the defense response and they interact in a homo and hetero combination. Many biotic stress resistance related proteins, including AtPR1 and AtPR2, were up-regulated in the double mutant of *AtCAX1* and *AtCAX3*, *atcax1/*3. These data suggested that the AtCAX1/AtCAX3 heterodimeric complex plays a role in defense response. Additionally, the AtCAX1/AtCAX3 complex is functionally involved in controlling the opening of stomata for maintaining the Ca^2+^ response and downstream signaling (Hocking et al., 2017). The *atcax1/3* mutant exhibited cell death at leaf tips and was compensated by lower external Ca^2+^ availability like *atcncg2/4*. It was suggested that CAXs function in plant disease resistance through the sequestration of Ca^2+^ to vacuoles (Shigaki et al., 2002; Cheng et al., 2003, 2005; Manohar et al., 2011b; Tian et al., 2020).

Some CAXs are known to be involved in transporting trace metal ions such as Mn^2+^, Cd^2+^ as well as Ca^2+^. CAXs broadly contribute to abiotic stress tolerance in plants, Specifically, in halophytic plants, CAXs mediate salt and heavy metal tolerance (Pittman and Hirschi, 2016). In mesophyll cells, CAXs are involved in stomatal conductance along with Ca^2+^ homeostasis. When the activities of CAXs were altered, the sensitivity of plants to metal stresses was altered (Bradshaw, 2005; Conn et al., 2011; Pittman and Hirschi, 2016). The double mutant of *AtCAX1* and *AtCAX3*, *atcax1/3*, showed the alteration of Ca^2+^ homeostasis, sensitivities to metals and tolerance to phosphate deficiency (Liu et al., 2018). CAXs are involved in not only salt stress, but also other abiotic stresses such as drought, cold, and heat stress. In accordance with these observations, their overexpression in various plant species resulted in an increased tolerance to abiotic stresses as well (Cheng et al., 2003, 2005; Zhao et al., 2009; Shigaki et al., 2010; Conn et al., 2011; Wu et al., 2011). *AtCAX1*, *AtCAX3*, *Glycine max* (soybean) *GmCAX1*, rice *OsCAX4*, *Malus* x *domestica* (apple) *MdCAXs* and *Gossypium hirsutum* (cotton) *GhCAX1* were transcriptionally regulated by abiotic stress and are known to function in process of cold acclimation with key roles related to salt, drought and freezing tolerance (Hirschi, 1999; Cheng et al., 2005; Kim et al., 2005; Manohar et al., 2011b; Han et al., 2012; Kamiya et al., 2012; Xu et al., 2013; Mao et al., 2021). The knockout of *AtCAX3* led to an increase in salt tolerance, but the *AtCAX1* knockout plants exhibited the freezing tolerance. Taken together, these observations suggest that there may be the different CAXs that are in charge of each stress response for maintaining [Ca^2+^]_cyt_ homeostasis (Bradshaw, 2005; Zhao et al., 2008, 2009; Bose et al., 2011). AtCAX11 performs critical roles for maintaining [Ca^2+^]_cyt_ homeostasis during hypoxia in roots and resulted in tolerance to water logging stress (Wang et al., 2016). The expression of tonoplast localized OsCCX2 increased under drought and salt stress, and was up-regulated by ABA but downregulated by Ca^2+^ deficiency. OsCCX2 in Ca^2+^ sensitive yeast mutant line resulted in the tolerance to zinc, iron and cobalt stress conditions (Yadav et al., 2015). In addition, Arabidopsis CAXs were revealed to be functionally involved in the response to boron deficiency (Quiles-Pando et al., 2013; Gonzalez-Fontes et al., 2014). AtCAX3 contributes to the sequestration of the boron-deficiency induced [Ca^2+^]_cyt_ to the vacuole (Gonzalez-Fontes et al., 2013; Quiles-Pando et al., 2013).

Heavy metal, cadmium-induced [Ca^2+^]_cyt_ was predominantly mediated by CAXs. AtCAX1, AtCAX3, and AtCAX4 contribute to Cd transport in a Cd stress condition and led to Cd tolerance (Wu et al., 2011, 2020; Baliardini et al., 2015; Ahmadi et al., 2018; Modareszadeh et al., 2021). However, these results still need to be confirmed to better understand whether they resulted from direct transport of metals by CAXs ions or by an alteration of a stress tolerance pathway by CAXs *via* maintaining [Ca^2+^]_cyt_ homeostasis.

## Conclusion and future perspective

In this review, recent findings pertaining to involvement of Ca^2+^ transport in biotic and abiotic stress was summarized, with a focus on Ca^2+^ influx and efflux. Overall, up-to-date research on Ca^2+^ signaling has led to significant progress, which have been enabled by cutting edge genomic tools. With the development of Ca^2+^ detection methods and recording techniques, long-awaited breakthroughs on Ca^2+^ signaling in plants will lead to advances in the effective management of plant stress.

Therefore, how these channels and pumps work together or independently to encode the specific Ca^2+^ signatures to various stress should be intensively explored. Another challenge to be addressed involves the identification and causal interconnection of Ca^2+^ channels for influx and counteracting Ca^2+^ pumps for efflux in relation to stress response. Restoration of the basal [Ca^2+^]_cyt_ levels is essential to terminate Ca^2+^ signaling and to reload Ca^2+^ stores. Therefore, sophisticated and coordinated regulation of Ca^2+^ channels and pumps should be addressed. An arising important question is how the limited numbers of Ca^2+^ channels encode a large number of Ca^2+^ signatures in plant cells. Many Ca^2+^ channel families have extended numbers of members and it is possible that they may also form hetero multimers. Studies on Arabidopsis CNGC and GLR members have provided the evidence that these channels interact with each other as a subunit and assemble into heteromeric functional Ca^2+^ channels. Therefore, it could be a general rule to generate a large repertoire of Ca^2+^ channels with heteromeric subunits in plants, encoding a large number of Ca^2+^ signatures involved in a wide array of biotic and abiotic stress processes.

## Author contributions

C-JP and RS wrote the manuscript and drew the diagram. All authors contributed to the article and approved the submitted version.

## Funding

This work was supported by a National Research Foundation of Korea (NRF) grant funded by the Korea government (MSIT) (NRF-2020R1A2C1007778 to C-JP) and a RIKEN CSRS Innovative Plant Biotechnology Collaboration Project (RIKEN CSRS to RS).

## Conflict of interest

The authors declare that the research was conducted in the absence of any commercial or financial relationships that could be construed as a potential conflict of interest.

## Publisher’s note

All claims expressed in this article are solely those of the authors and do not necessarily represent those of their affiliated organizations, or those of the publisher, the editors and the reviewers. Any product that may be evaluated in this article, or claim that may be made by its manufacturer, is not guaranteed or endorsed by the publisher.
